# Bioaccessibility and Gut Metabolism of Free and Melanoidin-Bound Phenolic Compounds From Coffee and Bread

**DOI:** 10.3389/fnut.2021.708928

**Published:** 2021-07-26

**Authors:** Genilton Alves, Leandro Araújo Lobo, Regina Maria Cavalcanti Pilotto Domingues, Mariana Monteiro, Daniel Perrone

**Affiliations:** ^1^Laboratório de Bioquímica Nutricional e de Alimentos, Biochemistry Department, Chemistry Institute, Federal University of Rio de Janeiro, Rio de Janeiro, Brazil; ^2^Laboratório de Biologia de Anaeróbios, Medical Microbiology Department, Paulo de Goés Microbiology Institute, Federal University of Rio de Janeiro, Rio de Janeiro, Brazil; ^3^Laboratório de Alimentos Funcionais, Nutrition Institute, Federal University of Rio de Janeiro, Rio de Janeiro, Brazil

**Keywords:** chlorogenic acids, ferulic acid, gut fermentation, maillard reaction, melanoproteins, melanosaccharides, simulated digestion

## Abstract

The aim of this study is to investigate the bioaccessibility and gut metabolism of free and melanoidin-bound phenolic compounds from coffee and bread. Phenolics from coffee were predominantly found in free forms (68%, mainly chlorogenic acids), whereas those from bread were mostly bound to melanoidins (61%, mainly ferulic acid). Bioacessibility of coffee total free phenolics slightly decreased during simulated digestion (87, 86, and 82% after the oral, gastric, and intestinal steps, respectively), with caffeoylquinic acids being isomerized and chlorogenic acids being partially hydrolyzed to the corresponding hydroxycinnamic acids. Bioacessibility of bread total free phenolics decreased during simulated digestion (91, 85, and 67% after the oral, gastric, and intestinal steps, respectively), probably related to complexation with the proteins in simulated gastric and intestinal fluids. Upon gut fermentation, the bioaccessibility of total free phenolics from both coffee and bread decreased, mainly after the first 4 h (56 and 50%, respectively). Caffeic and ferulic acids were the predominant metabolites found during coffee and bread gut fermentation, respectively. Melanoidin-bound phenolics from coffee and bread were progressively released after the gastric and intestinal steps, probably due to hydrolysis caused by the acidic conditions of the stomach and the action of pancreatin from the intestinal fluid. The bioaccessibilities of all phenolics from coffee and bread melanoidins after the gastric and intestinal steps were, on average, 11 and 26%, respectively. During gut fermentation, phenolics bound to both coffee and bread melanoidins were further released by the gut microbiota, whereas those from coffee were also metabolized. This difference could be related to the action of proteases on melanoproteins during gastrointestinal digestion, probably anticipating phenolics release. Nevertheless, bioaccessibilities of melanoidin-bound phenolics reached maximum values after gut fermentation for 24 h (50% for coffee and 51% for bread). In conclusion, the bioaccessibilities of coffee and bread free phenolics during simulated digestion and gut fermentation were remarkably similar, and so were the bioaccessibilities of coffee and bread melanoidin-bound phenolics.

## Introduction

In recent years, there has been a growing awareness regarding the beneficial effects of food on health. Current evidence strongly supports that diets rich in plant foods are associated with reduced risk of chronic diseases, including cardiovascular disease and type II diabetes (1, 2). These effects are mainly attributed to the presence of various bioactive compounds in these foods, such as phenolic compounds. These compounds are widely diffused in all plant foods including fruits, vegetables, and beverages, such as tea and coffee. Phenolics dietary intake ranges from 377.5 to 1365.1 mg per day, depending on the diet (3). Phenolics in foods occur in free forms (or soluble) or covalently bound to other molecules (or insoluble), especially cell wall components, such as cellulose, hemicellulose, pectin, and structural proteins (4).

In thermally processed foods, several studies have reported that phenolic compounds are involved in the formation of melanoidins, which are generically defined as high molecular weight nitrogenous and brown-colored molecules that contribute to food texture, color, and flavor (5). Melanoidins may be divided into two classes, melanosaccharides (skeletons composed mainly of polysaccharides), present in beverages such as coffee and beer, and melanoproteins (skeletons composed mainly of proteins), present in bakery products (6, 7). Although some studies report deleterious effects of melanoidins on health (5), other studies report that the incorporation of phenolic compounds into their structure present health benefits (8, 9). Phenolic compounds end up being bound to the backbones of melanoidins, as observed in coffee (3, 10–12), bread (3, 13), beer (3, 14), and many other commonly consumed food (3, 7, 15, 16). It is worth noting that theses melanoidin-bound phenolics are not accounted for in the estimates of dietary intake, causing their underestimation by up to 7% (3). However, the biological relevance of melanoidin-bound phenolics is still poorly understood. To exert their bioactivity, these compounds would have to be released from melanoidins prior to their absorption.

Although clinical studies are considered to be the best approach to investigate the metabolism of phenolic compounds (17, 18), *in vitro* simulated gastrointestinal digestion followed by gut fermentation have been used as a simpler, faster, and less expensive alternative (19–22). Recently, Pérez-Burillo et al. (23) reported the release of phenolics after simulated digestion and gut fermentation of melanoidins from different food sources. Moreover, Liu et al. (9) observed the cellular uptake and trans-enterocyte transport of the phenolics from digested vinegar melanoidins. Together, these studies indicate that melanoidin-bound phenolics have potential to exert biological effects. However, there are no studies in the literature investigating the step-by-step simulated digestion and gut fermentation of melanoidins. Moreover, the bioaccessibility of melanoidin-bound phenolics has never been determined, as well as their comparison to the bioaccessibility of free phenolics from the same food matrix. Thus, the aim of this study is to investigate the bioaccessibility and gut metabolism of free and melanoidin-bound phenolic compounds from coffee and bread.

## Materials and Methods

### Standards and Chemicals

Pepsin from porcine stomach mucosa (250 U/mg), pancreatin (8 × USP) from porcine pancreas, porcine bile extract, mucin from porcine stomach-type II, albumin, resazurin, cysteine, peptone, yeast extract, pectin, xylan, gum arabic, potato starch, casein, glucose, and inulin were purchased from Sigma-Aldrich Chemical Co. (St. Louis, MO, USA). Phenolic compounds standards (2,4 dihydroxybenzoic, 3,4 dihydroxybenzoic, gallic, benzoic, caffeic, ferulic, *p*-coumaric, salicylic, rosmarinic, and 5-caffeoylquinic acids) were purchased from Sigma-Aldrich Chemical Co. All solvents were HPLC grade from Tedia (Fairfield, OH, USA). HPLC grade water (Milli-Q system, Millipore, Bedford, MA, USA) was used throughout the experiments.

### Isolation of Free and Melanoidin-Bound Phenolic Compounds From Coffee and Bread

Coffee brew was prepared at 10% (*w*/*v*) by adding boiling water to commercial ground roasted coffee, agitating for 1 min using a magnetic stirrer (Corning TM, PC-220) with sufficient speed for complete mixing and filtering through a paper filter (Whatman n°. 1). Bread was produced in a domestic bread machine (BK2000B, Breadman, Middleton, WI) by adding in the following order: 245 mL of water, 15 mL of extra virgin olive oil, 12 g of brown sugar, 3 g of salt (NaCl), 30 g of wheat bran, 270 g of whole-wheat flour, and 3 g of dry yeast (*Saccharomyces cerevisiae*). The bread making process consisted of three stages: mixing (30 min), fermentation (116 min), and baking (47 min). Bread crusts were removed, freeze-dried (Labconco, Kansas City, MO), and ground in a mill (A11 Basic, IKAÒ Werke, Staufen, Germany). Extraction of bread crust melanoidins followed the procedure described by Borrelli et al. (24) with modifications. Briefly, 21 g of freeze-dried bread crust was mixed with 250 mL of 0.2 M Tris–HCl buffer (pH 8.0) containing 10 mg/mL of pancreatin from porcine pancreas. The solution was incubated in an orbital shaker (IKA KS 4000i control, Staufen, Germany) at 37°C for 70 h. After centrifugation (3,000 x *g*, 10 min, 25°C), the supernatant was collected.

The coffee brew and the supernatant containing bread melanoidins were subjected to ultrafiltration process using a submerged module system (PAM membranas, Rio de Janeiro, Brazil) with a polyethylenesulfone membrane (average pore size of 10 kDa; area of 12 cm^2^) working at a transmembrane pressure of 3 bar. The process was performed at 25°C, with a continuous agitation. The volume of permeate material (containing free phenolic compounds) was measured daily and an equivalent volume of water was added to the system.

The isolation of coffee free and melanoidin-bound phenolic compounds was carried out in two steps. In the first step, the contents of phenolics in the permeate reduced from 6913.4 mg/L (day 1) to 1492.8 mg/L (day 29). Then, the retentate was freeze-dried, resuspended in water, and subjected to a second ultrafiltration process, in which, after 21 days, the permeate did not present phenolics. The isolation of bread free and melanoidin-bound phenolic compounds followed the same procedure, but only one step, which lasted 23 days, was needed for obtaining a permeate with no phenolics present. Both permeate and retentate fractions were freeze-dried, yielding materials containing free and melanoidin-bound phenolics, respectively.

### Analysis of Free and Melanoidin-Bound Phenolic Compounds

Phenolic compounds analysis in coffee and bread samples followed the methodology described by Perrone, Farah, and Donangelo (11), with modifications. For free phenolics, both permeate and retentate samples (1 mg/mL in water) were mixed with solutions of Carrez (0.3 M K_2_Fe (CN)_6_ and 1.0 M zinc acetate) (1:100 *v*/*v*) for clarification and filtered through a 0.45 μm cellulose ester membrane (Millipore®, São Paulo, Brazil) prior to HPLC-DAD analysis. For melanoidin-bound phenolics, retentate samples were subjected to alkaline hydrolysis. Briefly, an aliquot of 750 μL (10 mg/mL in water) was mixed with 750 μL of 2 M NaOH solution containing 2% (*w*/*w*) ascorbic acid and 20 mM ethylenediaminetetraacetic acid (EDTA). After incubation for 1 h at 30°C, hydrolysis was interrupted by adjusting the pH to 1 with 330 μL of 5 M HCl, clarification was performed by adding 20 μL of each solutions of Carrez and 130 μL of water and the sample was centrifuged prior to HPLC-DAD analysis.

The LC system (Shimadzu, Kyoto, Japan) comprised a LC- 10ADvp quaternary pump, a CTO-10ASvp column oven, an 8125 manual injector (Rheodyne) with a 20 μL loop, and an SPD-M10Avp diode array detector (DAD). Free and melanoidin-bound phenolics in coffee were analyzed according to the method described by Farah et al. (25). Chromatographic separations were achieved using a Magic C30 HPLC column (150 2 mm, 5 μm, 100 A, Michrom Bioresources, Inc., Auburn, CA, USA) maintained at a constant temperature of 40°C. The LC two-phase mobile system consisted of 0.3% aqueous formic acid (eluent A) and methanol (eluent B). The gradient was programmed with a flow rate of 0.3 mL/min.

To investigate the content of free and melanoidin-bound phenolics in bread were analyzed according to the method described by Alves and Perrone (13). Chromatographic separations were achieved using a Kromasil® C18 column (5 μm, 250 mm, 4.6 mm) maintained at a constant temperature of 40°C. The LC mobile system consisted of a gradient of water with 0.3% formic acid (eluent A), methanol (eluent B), and acetonitrile (eluent C, kept at 1% during the whole run), with a constant flow rate of 1.0 mL/min.

Phenolic compounds were monitored by DAD between 190 and 370 nm and identified by comparison of their retention times and UV spectra with those of commercial standards. Quantification was performed by external standardization. The quantification of caffeoylquinic acids (CQA), feruloylquinic acids (FQA), and di-caffeoylquinic acids (diCQA) was performed using the diode array data for the peak area of 5-CQA standard corrected with molar extinction coefficients of the respective CGA, as previously described by Farah et al. (25). Data were acquired by LCMS solution software (Shimadzu Corp., version 2.00, 2000). Results were expressed as μg/g.

### *In vitro* Gastrointestinal Digestion and Gut Fermentation of Free and Melanoidin-Bound Phenolics

*In vitro* gastrointestinal digestion and gut fermentation were performed to mimic human physiological conditions during oral, gastric, small intestinal, and gut steps. The study was conducted following the ethical principles involving human subjects defined in the Declaration of Helsinki and was approved by the research ethics committee of Clementino Fraga Filho Hospital from Federal University of Rio de Janeiro, Brazil (approval number 512.847). The subjects signed an informed consent form. The bioaccessibility of each phenolic compound was calculated as the ratio between the molar concentrations, adjusted to the number of phenolic acid moieties in the molecule (e.g., CQA contains one, whereas di-CQA contains two), in each digestion phase and in the corresponding samples.

#### In Vitro Gastrointestinal Digestion

The *in vitro* gastrointestinal digestion was performed as described by Fernández and Labra (26) and later by de Almeida et al. (19). Three parallel experiments, each reaching a given step of the digestion (oral, gastric, and intestinal) were performed in triplicate. In a glass vial, an aliquot of 0.5 g of freeze-dried coffee and bread samples were mixed with 3 mL of human saliva and 2 mL of water, and the mixture was incubated at 37°C in an orbital shaker (Sorvall ST 16R, Thermo Scientific^TM^) for 1 min at 260 rpm. After the oral step, 2.5 mL of simulated gastric fluid (Supplementary Table 1) was added, and the pH was adjusted to 2.0 with 5 M HCl. Then, vials were sealed with a silicone septum and atmospheric air was replaced by introducing gaseous nitrogen. Gastric digestion was performed by incubating the mixture at 37°C in an orbital shaker for 2 h at 260 rpm. After the gastric step, vials were opened, 2 mL of simulated intestinal fluid (Supplementary Table 1) were added, and the pH was adjusted to 6.5 with 1 M NaHCO_3_. Vials were resealed and nitrogen atmosphere was reestablished. Intestinal digestion was performed by incubating the mixture at 37°C in an orbital shaker for 2 h at 260 rpm.

Samples obtained after oral, gastric, and intestinal steps were centrifuged (3,000 × *g*, 15 min, 25°C) and the supernatants were passed through Amicon® centrifugal filters with a 10 kDa membrane cut-off (Millipore, Cork, Ireland) prior to HPLC-DAD analysis, as described in Section 2.3.

#### In Vitro Gut Fermentation

Gut fermentation was performed following the methodology of Inada et al. (20). Fresh feces were donated by a healthy male subject (31 years old, BMI of 24.7 kg/m^2^) that had regular bowel function, no gastrointestinal diseases, and that did not use antibiotics, dietary supplements, probiotics, prebiotics, and symbiotics in the 3 months prior to the study. For 48 h prior to feces collection, the subject followed a phenolic-free diet (avoiding fruits and vegetables, legumes, whole cereals, and beverages such as coffee, tea, maté, fruit juices, soymilk) and also did not consume yogurts and alcoholic beverages.

In glass test tubes, fecal slurries were diluted with culture growth medium (Supplementary Table 1) at 5% and mixed with the sample from the last step of the gastrointestinal digestion, which contained all the digested material. Fecal fermentation was performed by incubating the mixture at 37°C in an anaerobic chamber (Coylabs, USA) with an atmosphere containing 10% H_2_, 10% CO_2_, and 80% N_2_ under orbital shaking at 50 rpm for 4, 24, and 48 h. Blank experiments in which the digested samples were not added to the fecal slurry were performed to account for the presence of phenolics in the feces samples. In these experiments, no phenolic compounds were detected, indicating that the dietary restriction was adequate to ensure their absence from feces. Parallel experiments, one for each fermentation time, were performed in triplicate. Samples were centrifuged (3,000 × g, 15 min, 25°C), the supernatant was sequentially filtered through 0.45 μm and 0.22 μm cellulose ester membranes (Analitica, São Paulo, Brazil), and were passed through Amicon® centrifugal filters with a 10 kDa membrane cut-off (Millipore, Cork, Ireland) prior to HPLC-DAD analysis, as described in Section “Analysis of free and melanoidin-bound phenolic compounds.”

### Statistical Analysis

Data were expressed as mean ± standard deviation and processed using Prism for Windows software, version 8.01 (GraphPad Software Inc.). Analysis of variance (one-way ANOVA) followed by Tukey's *post hoc* test was used to compare the contents of phenolic compounds released among simulated digestion phases (oral, gastric, intestinal, and gut fermentation). Differences were considered significant when *p* < 0.05.

## Results and Discussion

### Phenolics From Coffee Were Predominantly Found in Free Forms Whereas Those From Bread Were Mostly Bound to Melanoidins

Three CQA isomers (3-CQA, 4-CQA, and 5-CQA), three FQA isomers (3-FQA, 4-FQA, and 5-FQA) and three diCQA isomers (3,4-diCQA, 3,5-diCQA, and 4,5-diCQA) were identified among coffee free phenolics (Table 1). 5-CQA was the most abundant CGA (27%), followed by 3-CQA (23%) and 4-CQA (18%), which is in agreement with the literature (27, 28). Caffeic, ferulic, 3,4-dihydroxybenzoic, gallic, and salicylic acids were identified among coffee melanoidin-bound phenolics (Table 1). Hydroxycinnamic acids (caffeic and ferulic acids) were the most abundant (67%), being related to the incorporation of CGA (hydroxycinnamic acid esters) into melanoidins backbone during coffee roasting (10, 12, 29). Hydroxybenzoic acids represented the remaining phenolics, being 3,4-dihydroxybenzoic and gallic acids the most abundant (69% in total). These compounds have already been identified in coffee melanoidin samples (29–31). In coffee, total free phenolics contents (11,129.3 μg/g) were higher than that of total melanoidin-bound phenolics (5,278.5 μg/g), as expected from literature data (10, 25). One should consider that phenolics are bound to coffee melanoidin through ester bonds, as well as condensed structures and glycosidic bonds. In the present work, we choose to employ only alkaline hydrolysis to analyze coffee melanoidins and, therefore, only phenolic compounds linked through ester bonds were taken into account.

**Table 1:** Free and melanoidin-bound phenolic compounds in coffee.

**Phenolic compound**	**Content (μg/g)^***a***^**
***Free phenolics (permeate fraction)***	
3-Caffeoylquinic acid	2,532.2
4-Caffeoylquinic acid	2,014.1
5-Caffeoylquinic acid	2,980.3
3-Feruloylquinic acid	739.3
4-Feruloylquinic acid	615.2
5-Feruloylquinic acid	944.5
3,4-Dicaffeoylquinic acid	456.6
3,5-Dicaffeoylquinic acid	339.9
4,5-Dicaffeoylquinic acid	507.2
**Total free phenolics**	**11,129.3**
***Melanoidin-bound phenolics (retentate fraction)***	
Caffeic acid	2,312.7
Ferulic acid	1,226.4
3,4-Dihydroxybenzoic acid	607.2
Gallic acid	596.8
Salicylic acid	535.4
**Total melanoidin-bound phenolics**	**5,278.5**

a *Results expressed as mean of two replicates; Coefficient of variation lower than 10% for all phenolic compounds*.

Gallic, caffeic 3,4-dihydroxybenzoic, ferulic, and rosmarinic acids were identified among bread free phenolics. The first three compounds were the most abundant, corresponding to 78% of free phenolics (Table 2). With the exception of rosmarinic acid, these phenolic compounds identified were linked to bread melanoidins, with the addition of 2,4-dihydroxybenzoic acid. The profile in this sample, however, was very different, with ferulic acid corresponding to 61% of bound phenolics (Table 2), as widely reported in the literature (32, 33). All these phenolics have already been reported in whole-grain wheat flour and bread (13, 19, 34, 35). Contrary to coffee, melanoidin-bound phenolics were more abundant (2,294.5 μg/g) in bread than free phenolics (1,452.5 μg/g), in accordance with previous reports (35–38).

**Table 2:** Free and melanoidin-bound phenolic compounds in bread.

**Phenolic compound**	**Content (μg/g)^***a***^**
**Free phenolics (permeate fraction)**	
Gallic acid	448.2
Caffeic acid	367.7
3,4-Dihydroxybenzoic acid	315.2
Ferulic acid	190.7
Rosmarinic acid	130.2
**Total free phenolics**	**1,452.0**
**Melanoidin-bound phenolics (retentate fraction)**	
Ferulic acid	1,397.0
2,4-Dihydroxybenzoic acid	605.7
Caffeic acid	119.9
Gallic acid	93.4
3,4-Dihydroxybenzoic acid	78.5
**Total melanoidin-bound phenolics**	**2,294.5**

a *Results expressed as mean of two replicates; Coefficient of variation lower than 10%*.

### Free Phenolic Compounds From Coffee and Bread Were Extensively Metabolized During Gastrointestinal Digestion and Gut Fermentation

#### Coffee

**Figure 1:**
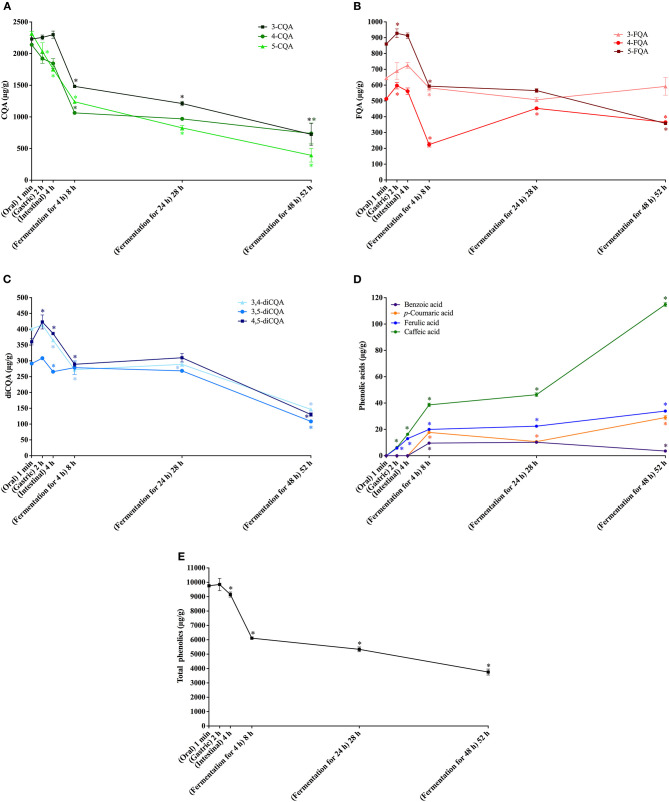
*In vitro* simulated digestion (oral, gastric and intestinal steps) and gut fermentation of coffee free phenolic compounds: caffeoylquinic acids (CQA, **A**), feruloylquinic acids (FQA, **B**), dicaffeoylquinic acids (diCQA, **C**), phenolic acids **(D)** and total phenolics **(E)**. The asterisk indicates a significant difference in relation to the previous step (ANOVA followed by Tukey's post-test, *p* < 0.05).

All CGA previously found in coffee permeate material (containing free phenolics) were identified after the oral digestion step, that is, three CQA isomers, three FQA isomers, and three di-CQA isomers (Figures 1A–C). On average, free phenolics bioaccessibility after oral digestion was 87% (Table 3), indicating their almost complete dissolution in saliva. CQA and FQA showed higher bioaccessibilities (on average 89 and 88%, respectively) than diCQA (on average 81%), which may be explained by the lower water solubility and higher lipophilicity of the latter class in comparison to the former ones.

**Table 3:** Bioaccessibility (%) of free and melanoidin-bound phenolic compounds from coffee during simulated digestion (oral, gastric, and intestinal steps) and gut fermentation.

**Compound**	**Oral %_*material^a^*_**	**Gastric**		**Intestinal**		**Gut fermentation**					
						**4 h**		**24 h**		**48 h**	
		**%_**material**_**	**%_previous^b^_**	**%_**material**_**	**%_**previous**_**	**%_**material**_**	**%_**previous**_**	**%_**material**_**	**%_**previous**_**	**%_**material**_**	**%_**previous**_**
*Free phenolics*											
3-Caffeoylquinic acid	88	89	101	91	102	59	64	48	82	29	60
4-Caffeoylquinic acid	106	95	90	92	96	53	58	48	91	37	76
5-Caffeoylquinic acid	78	68	88	59	86	42	71	28	67	13	48
3-Feruloylquinic acid	87	93	107	98	105	79	80	69	87	80	117
4-Feruloylquinic acid	83	97	116	91	94	36	40	74	202	59	81
5-Feruloylquinic acid	91	98	108	97	98	63	65	60	95	38	63
3,4-Dicaffeoylquinic acid	88	91	103	80	88	60	75	63	106	32	51
3,5-Dicaffeoylquinic acid	86	91	106	78	86	82	105	79	96	32	40
4,5-Dicaffeoylquinic acid	71	83	117	76	91	57	75	61	107	26	42
Caffeic acid	-^*c*^	–	–	–	261	–	237	–	120	–	248
Ferulic acid	–	–	–	–	224	–	154	–	113	–	151
*p*-Coumaric acid	–	–	–	–	–	–	–	–	61	–	270
Benzoic acid	–	–	–	–	–	–	–	–	107	–	35
**Total phenolics**	**87**	**86**	**99**	**82**	**95**	**56**	**68**	**51**	**90**	**35**	**69**
*Melanoidin-bound phenolics*											
Caffeic acid	0	8	0	14	174	28	205	18	64	10	53
Ferulic acid	0	11	0	18	156	39	222	44	112	17	37
3,4-Dihydroxybenzoic acid	0	16	0	21	131	81	391	148	183	78	53
Gallic acid	0	21	0	33	157	107	326	75	70	105	141
Salicylic acid	0	15	0	56	382	28	50	42	149	63	149
**Total phenolics**	**0**	**12**	**0**	**23**	**194**	**46**	**199**	**50**	**107**	**38**	**76**

a *Calculated in relation to the content of phenolics in the initial material (see Table 1)*.

b *Calculated in relation to the content of phenolics in the previous digestion step or gut fermentation time*.

c *Not applicable*.

No change in coffee total free phenolics bioaccessibility was observed after the gastric digestion step. However, isomerization of CQA isomers was observed (Figure 1A). In comparison to the permeate material, 5-CQA relative content decreased from 40 to 33% at the same time that 3-CQA and 4-CQA relative contents increased from 34 to 36% and from 27 to 31%, respectively. In fact, acyl migration is known pathway of CGAs transformation. According to Farrell et al. (39), approximately 2% of 5-CQA is converted to 3-CQA and 4-CQA after 2 h of incubation at pH 7.4. In addition to isomerization, very low quantities of caffeic and ferulic acids were observed (Figure 1D), indicating hydrolysis (0.13% of CQA + diCQA to caffeic acid and 0.48% of FQA to ferulic acid). Even though CGA are said to be stable in artificial and natural gastric fluids (40), Lafay et al. (41) reported traces of caffeic acid in the stomach of rats fed with a diet supplemented with CGA. Considering that gastric juice do not possess esterases capable of hydrolyzing CGA to release caffeic and ferulic acids (42), we can suppose that in the stomach, CGA would be hydrolyzed to release the corresponding hydroxycinnamic acids due to the acidic conditions. After the intestinal step, coffee free phenolics bioaccessibility slightly decreased (Figure 1E), reaching 82% (Table 2). Moreover, isomerization of 5-CQA to 3-CQA continued to occur and CGA hydrolysis increased, with 0.34% of the sum of CQA and diCQA being converted to caffeic acid and 1.07% of FQA being hydrolyzed to ferulic acid. Both isomerization and hydrolysis of CGA in intestinal fluids have been reported in the literature (40).

After simulation of gastrointestinal digestion, coffee permeate material was submitted to gut fermentation. During 48 h of fermentation, a decrease in total phenolic compounds bioaccessibility was observed (Figure 1E). The most expressive reduction was observed after the first 4 h, reaching 56%, followed by reductions after 24 h (51%) and 48 h (35%) (Table 2). Caffeic acid was the metabolite found at the highest concentrations throughout the fermentation process. In fact, during gut fermentation, CGA continue to be hydrolyzed, especially toward the end of the 48 h, when 2.6% of CGA, on average, were converted to their corresponding hydroxycinnamic acids (Figure 1D). FQA showed a lower decrease in their bioaccessibility (from 96% at the intestinal step to 57% after 48 h of gut fermentation) in comparison to CQA and diCQA (from 78 to 28%), suggesting that the former class was less metabolized than the latter ones. The presence of benzoic acid may be explained by aromatization of quinic acid (43) or hydrolysis and β-oxidation of caffeic and ferulic acids (44).

Even though only 3.6% of free phenolics present in coffee permeate material were quantified as metabolites after gut fermentation for 48 h, the intense decrease in total CGA suggests their extensive metabolization, since they are known to be stable at these pH and temperature conditions. Even though Ludwig et al. (45) reported that dihydrocaffeic acid was the major metabolite of CGA formed during gut fermentation, we did not found this compound in our experiments. Also, differently from our results, these authors reported that caffeic and ferulic acids were initial degradation products, with a transient appearance and maximum quantities after 1 h of fermentation. These differences may result from the rate and extent of the degradation, which show a clear influence of amount and composition of the gut microbiota (46). In fact, Breynaert et al. (47) observed different outcomes when CGA were incubated for 6 h with two concentration of gut microorganisms. At the higher concentration (108 CFU/mL), CGA were completely metabolized, yielding mainly dihydrocaffeic acid. On the other hand, at a lower concentration (105 CFU/mL), CGA were still observed, and the main metabolite was caffeic acid. Therefore, we can suppose that the concentration of microorganisms in our fecal slurry was probably not sufficient to catabolize all CGA present.

#### Bread

**Figure 2:**
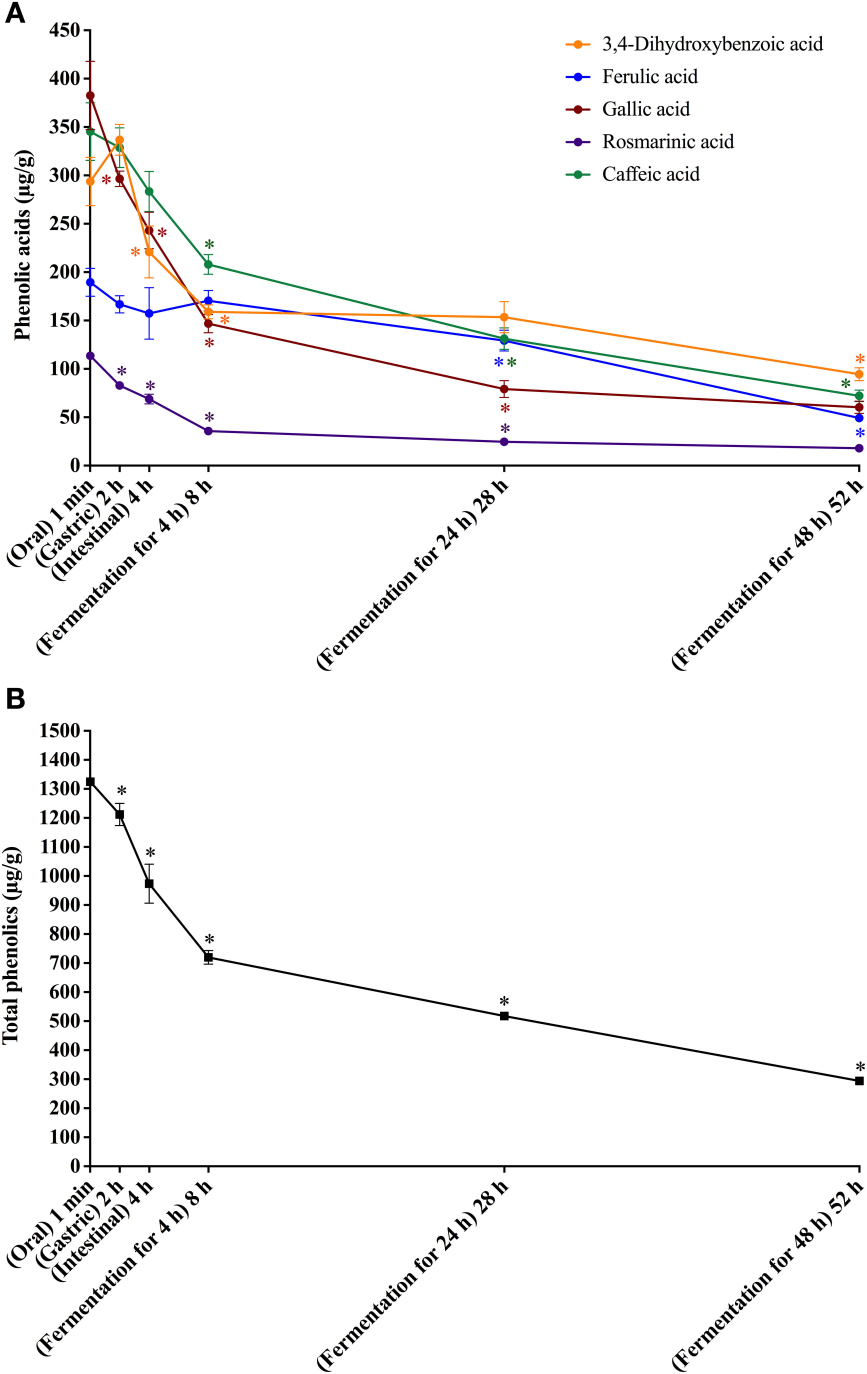
*In vitro* simulated digestion (oral, gastric and intestinal steps) and gut fermentation of bread free phenolic compounds: phenolic acids **(A)** and total phenolics **(B)**. The asterisk indicates a significant difference in relation to the previous step (ANOVA followed by Tukey's post-test, *p* < 0.05).

All phenolic compounds previously found in bread permeate material (containing free phenolics) were identified after the oral digestion step, that is three hydroxycinnamic acid derivatives (caffeic, ferulic, and rosmarinic acids) and two hydroxybenzoic acid derivatives (gallic and 3,4-dihydroxybenzoic acids) (Figure 2A). On an average, free phenolics bioaccessibility after oral digestion was 91% (Table 4), indicating their almost complete dissolution in saliva related to their high solubility in water.

**Table 4:** Bioaccessibility (%) of free and melanoidin-bound phenolic compounds from bread during simulated digestion (oral, gastric, and intestinal steps) and gut fermentation.

**Compound**	**Oral %_material^a^_**	**Gastric**		**Intestinal**		**Gut fermentation**					
						**4 h**		**24 h**		**48 h**	
		**%_**material**_**	**%_previous^b^_**	**%_**material**_**	**%_**previous**_**	**%_**material**_**	**%_**previous**_**	**%_**material**_**	**%_**previous**_**	**%_**material**_**	**%_**previous**_**
*Free phenolics*											
Gallic acid	85	66	78	54	82	33	60	18	54	13	76
Caffeic acid	94	89	95	77	86	57	73	36	63	20	55
3,4-Dihydroxybenzoic acid	93	107	115	70	66	50	72	49	97	30	62
Ferulic acid	96	87	91	83	94	89	108	68	76	26	38
Rosmarinic acid	87	64	73	53	83	27	52	19	69	14	73
**Total phenolics**	**91**	**85**	**93**	**67**	**79**	**50**	**74**	**36**	**73**	**21**	**57**
*Melanoidin-bound phenolics*											
Ferulic acid	0	9	0	36	396	36	103	55	152	44	79
2,4-Dihydroxybenzoic acid	0	7	0	17	234	19	110	42	219	57	135
Caffeic acid	0	25	0	50	196	59	119	86	145	51	59
Gallic acid	0	15	0	29	192	45	156	26	59	70	265
3,4-Dihydroxybenzoic acid	0	9	0	21	233	17	83	46	265	55	118
**Total phenolics**	**0**	**10**	**0**	**30**	**310**	**32**	**107**	**51**	**161**	**50**	**97**

a *Calculated in relation to the content of phenolics in the initial material (see Table 2)*.

b *Calculated in relation to the content of phenolics in the previous digestion step or gut fermentation time*.

*^c^Not applicable*.

After the gastric digestion step, the bioaccessibility of bread total free phenolics decreased (Figure 2B), reaching 85% (Table 4), as a consequence of the reduction in the contents of gallic and rosmarinic acids (Figure 2A). The free phenolic compounds may form low-solubility complexes with proteins (34), such as those from the simulated gastric juice. These complexes cannot pass through the filtration membrane used in the present study (17, 48), possibly explaining the observed reduction in bioaccessibility. This effect could not be observed in the experiments with coffee probably due to their much higher free phenolics content (11.1 mg/g) in comparison to bread (1.4 mg/g). After the intestinal step, bread free phenolics bioaccessibility continued to decrease (Figure 2B), reaching 67% (Table 4). This was caused by decrease in the contents of gallic, caffeic, and 3,4-dihydroxybenzoic acids, which were probably related to further complexation with the proteins present in the simulated intestinal fluid.

Upon gut fermentation, total phenolic compounds bioaccessibility progressively reduced (Figure 2B). As observed for coffee free phenolics, the most expressive reduction occurred after 4 h, reaching 50%, decreasing to 36 and 21% after 24 and 48 h, respectively (Table 4). Considering individual phenolics, the contents of all compounds decreased after 4 h of gut fermentation, with the exception of ferulic acid, which did not change. In the following times, the decreases were less pronounced, and it is worth noting that only 3,4-dihydroxybenzoic acid contents remained unaltered after 24 h of fermentation. This result may be explained by a possible conversion of gallic acid (3,4,5-trihydroxybenzoic acid) through the loss of hydroxyl in its structure (49). Together, the results of this study show that bread free phenolics were extensively metabolized during simulated digestion and gut fermentation, as also described above for coffee free phenolics.

### Bound Phenolic Compounds From Coffee and Bread Were Released From Melanoidins and Metabolized During Gastrointestinal Digestion and Gut Fermentation

**Figure 3:**
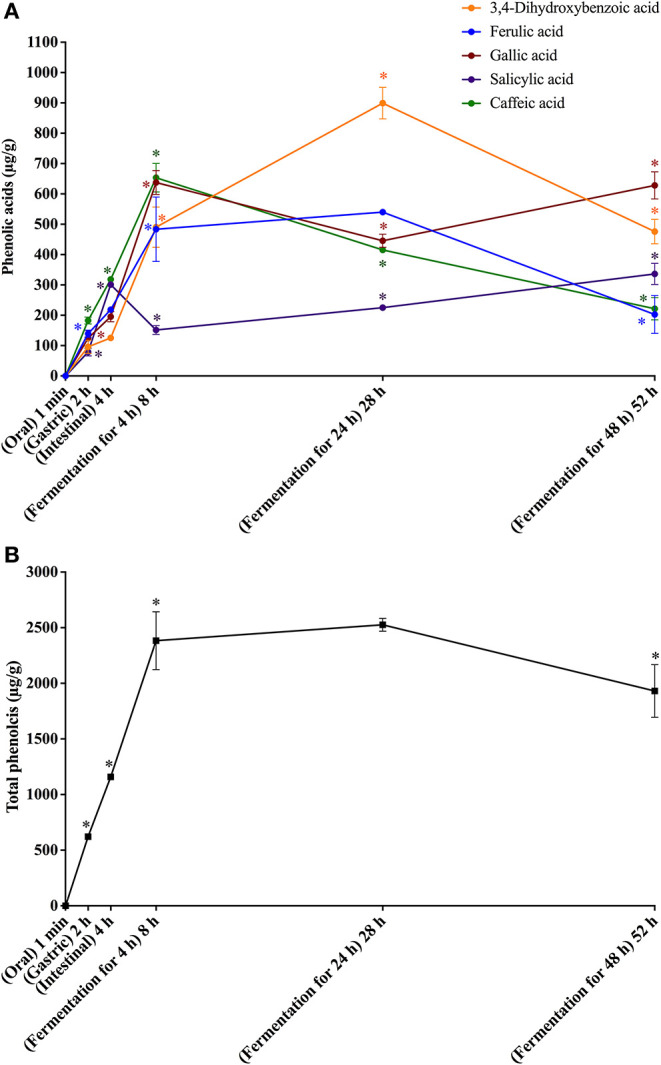
*In vitro* simulated digestion (oral, gastric and intestinal steps) and gut fermentation of coffee melanoidin-bound phenolic compounds: phenolic acids **(A)** and total phenolics **(B)**. The asterisk indicates a significant difference in relation to the previous step (ANOVA followed by Tukey's post-test, *p* < 0.05).

**Figure 4:**
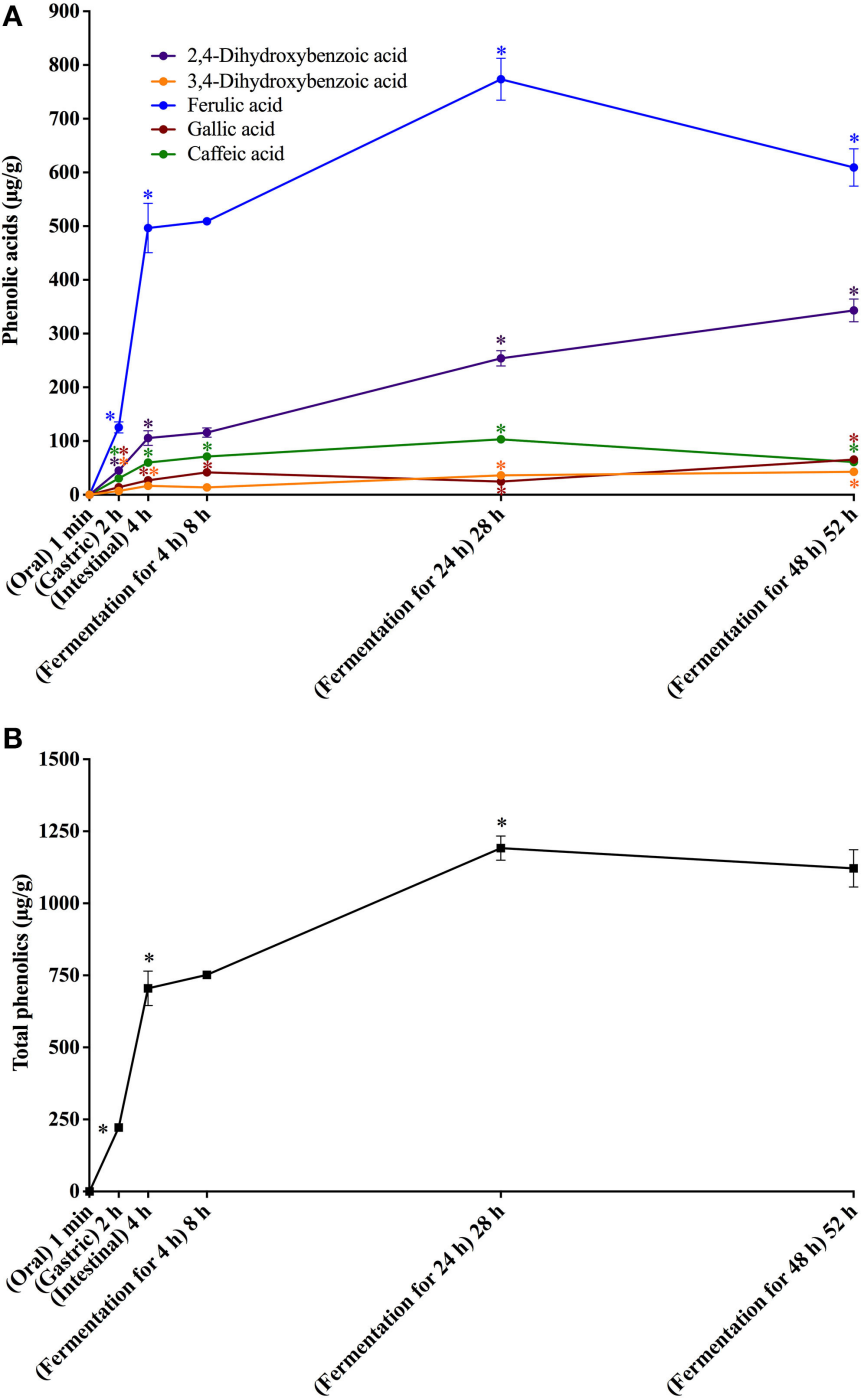
*In vitro* simulated digestion (oral, gastric and intestinal steps) and gut fermentation of bread melanoidin-bound phenolic compounds: phenolic acids **(A)** and total phenolics **(B)**. The asterisk indicates a significant difference in relation to the previous step (ANOVA followed by Tukey's post-test, *p* < 0.05).

Different from that observed after the oral digestion step of free phenolics, no phenolic compounds were observed after the oral digestion step of coffee and bread retentates (Figures 3A, 4A). This is probably related to the very short incubation time (1 min) and to the neutral pH, which were insufficient to release phenolics from the melanoidins skeleton. All phenolics previously found in coffee and bread retentate material (containing melanoidin-bound phenolics) were identified after the gastric digestion step: two hydroxycinnamic acid derivatives (caffeic and ferulic acids) and three hydroxybenzoic acid derivatives (3,4-dihydroxybenzoic, gallic, and salicylic acids) in coffee; two hydroxycinnamic acid derivatives (caffeic and ferulic acids) and three hydroxybenzoic acid derivatives (2,4-dihydroxybenzoic, 3,4-dihydroxybenzoic, and gallic acids) in bread. In comparison to the retentate materials, the bioaccessibility of all phenolics from coffee and bread melanoidins progressively increased (Figures 3B, 4B) after the gastric (12 and 10%, on average, respectively) and intestinal steps (23 and 30%, on average, respectively) (Tables 3, 4). This was due to the release of phenolics, which may be associated to the acidic conditions of the stomach, as well as the action of pancreatin in the intestinal fluid, which would hydrolyze the ester bonds that most commonly link phenolics to melanoidins (50). In addition, proteases in the gastric and intestinal fluids could act on melanoproteins, enhancing the action of the aforementioned esterases. This would possibly explain the higher phenolics release from bread in comparison to coffee, since the former is known to contain melanoproteins (51) and the latter melanosaccharides (7). In terms of individual compounds and comparing the intestinal with the gastric step, it is worth noting that salicylic acid was the phenolic with the most expressive release from coffee melanoidins, whereas the same can be said on ferulic acid from bread melanoidins (Figures 3A, 4A).

**Figure 5:**
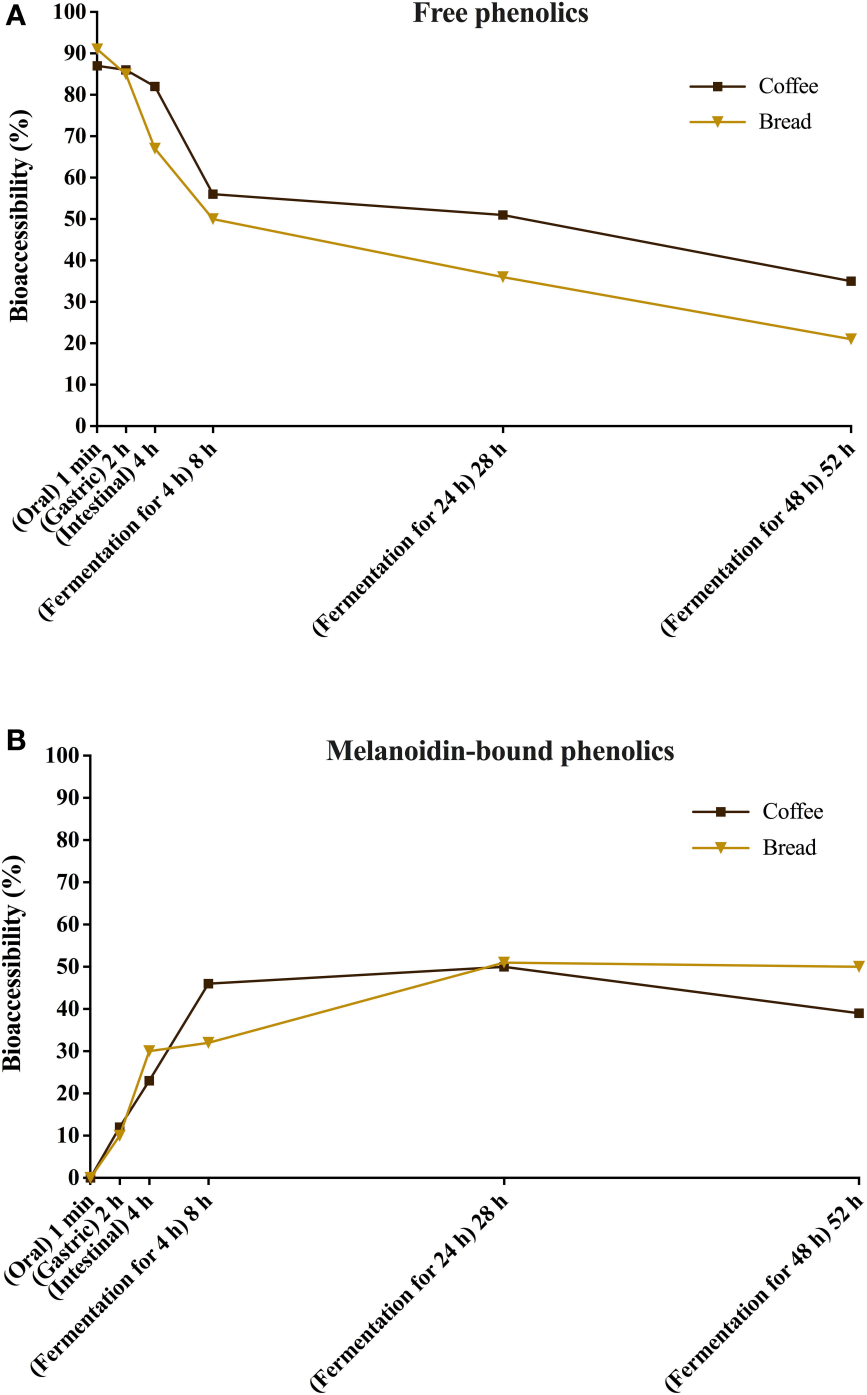
Bioaccessibility of total free **(A)** and total melanoidin-bound **(B)** phenolics from coffee and bread expressed as the percentage of compounds released during the simulated digestion and gut fermentation stages in relation to the total phenolics content in the corresponding sample.

Upon gut fermentation for 4 h, total phenolic compounds contents from coffee doubled (Figure 3A), indicating an extensive release from melanoidins due to the action of microbiota, leading to a bioaccessibility of 46% (Table 3). On the other hand, total phenolic compounds contents from bread did not change during the same period (Figure 3B), leading to a bioaccessibility of 32% (Table 4). This difference could be related to the action of proteases during gastrointestinal digestion, as previously discussed, which probably anticipated phenolics release. After 24 h of gut fermentation, phenolic compounds bioaccessibility of coffee bound–phenolics did not significantly change (50%) and decreased thereafter (38%) (Table 3). In contrast, phenolic compounds bioaccessibility of bread bound–phenolics increased after 24 h of gut fermentation (51%) and did not significantly change (50%) after 48 h (Table 4). These results indicate that while phenolics bound to both coffee and bread melanoidins were released by the gut microbiota, those from coffee were further metabolized (Figure 5).

Recently, Pérez-Burillo et al. (23) submitted melanoidins from different food sources, including coffee and bread, to simulated digestion and gut fermentation. According to these authors, gut microbes were able to release some phenolics initially linked to the melanoidin backbone. However, this study was the first one, to the best of our knowledge, to investigate the step-by-step effect of simulated digestion and gut fermentation on melanoidins and to determine the bioaccessibility of melanoidin-bound phenolics. The amounts of phenolics released from coffee and bread melanoidins were much higher in our study, respectively 2,525.9 μg/g and 1,191.9 μg/g after gut fermentation for 24 h, than those reported by Pérez-Burillo et al. (23), of 48.8 μg/g and 2.2 μg/g after gut fermentation for 20 h. These differences could be related to the different media used in the studies. While we carried out gut fermentation experiments with a nutrient-rich medium, considered to be more representative of the carbon sources physiologically available to microbiota, those authors used a nutrient-poor medium, which could artificially stimulate the use of phenolic compounds as sources of carbon skeletons. Moreover, the medium of this study is more appropriate to maintain the taxonomic diversity of the feces microbiota, as nutrient-poor media are known to impede the survival of many species of bacteria (52). Pérez-Burillo et al. (23) also discussed that coffee melanoidins released more phenolics than bread melanoidins, which was similar to the one observed in this study. However, when the amount of phenolics initially linked to melanoidins backbone is considered, we observed that the bioaccessibility of phenolic compounds from coffee and bread melanoidins was similar (50% and 51% after 24 h of gut fermentation, respectively) (Figure 5).

Phenolic compounds and their metabolites interact with the gut microbiota, exerting effects on human health. Many studies show that there is a bi-directional interaction between dietary polyphenols and the gut microbiota, being mutually beneficial (52). In the particular case of melanoidins, their digestion caused an increase in the production of short-chain fatty acids and favored the growth of beneficial microorganisms (23), possibly acting as prebiotics. In fact, many authors consider that melanoidins have characteristics of dietary fibers, being highly metabolized by the gut microbiota (53–55). In addition to these *in situ* effects, phenolics released from melanoidins could be potentially absorbed in the gut and exert several systemic biological activities (52). In this context, it is worth mentioning that considering that the daily intake of coffee melanoidins leads to the intake of 19.5 mg of phenolics (3), one could argue that these macromolecules would significantly contribute to the health effects of coffee consumption. Finally, we can hypothesize that our results regarding the release of bound phenolics could be extrapolated, at least in part, to non-thermally processed plant foods. In cereal-based foods, most phenolic compounds are found in the insoluble bound form, while in fruits and vegetables their content is lower, but still considerable (56, 57). If this hypothesis proves true, it would be undeniable that insoluble phenolics play a key role in the health effects associated with diets rich in fruits, vegetables, and especially whole grains.

## Conclusions

Phenolics from coffee were predominantly found in free forms, whereas those from bread were mostly bound to melanoidins. During simulated digestion of coffee free phenolics, CQA isomerized and CGA hydrolyzed forming the corresponding hydroxycinnamic acids. Bioacessibility of bread total free phenolics decreased during simulated digestion, probably related to complexation with the proteins in simulated gastric and intestinal fluids. Upon gut fermentation, the bioaccessibility of total phenolic compounds from both coffee and bread decreased, mainly after the first 4 h. Caffeic and ferulic acids were the predominant metabolites found during coffee and bread gut fermentation, respectively. Melanoidin-bound phenolics from coffee and bread were progressively released after the gastric and intestinal steps, probably due to hydrolysis caused by the acidic conditions of the stomach and the action of pancreatin from the intestinal fluid. During gut fermentation, phenolics bound to both coffee and bread melanoidins were further released by the gut microbiota, whereas those from coffee were also metabolized. This difference could be related to the action of proteases on melanoproteins during gastrointestinal digestion, probably anticipating phenolics release. Finally, the bioaccessibilities of coffee and bread free phenolics during simulated digestion and gut fermentation were remarkably similar, and so were the bioaccessibilities of coffee and bread melanoidin-bound phenolics. Further analyses should be done in the future using high-resolution mass spectrometry to describe in more detail the gut metabolites from phenolics released from melanoidins.

## Data Availability Statement

The raw data supporting the conclusions of this article will be made available by the authors, without undue reservation.

## Ethics Statement

The studies involving human participants were reviewed and approved by Research ethics committee of Clementino Fraga Filho Hospital from Federal University of Rio de Janeiro, Brazil (approval number 512.847). The patients/participants provided their written informed consent to participate in this study.

## Author Contributions

GA: Methodology, validation, formal analysis, investigation, data curation, writing–original draft, visualization. LL and RD: Resources, writing–review and editing. MM: Writing–review and editing, visualization. DP: Conceptualization, data curation, writing–review & editing, visualization, supervision, project administration, funding. All authors contributed to the article and approved the submitted version.

## Conflict of Interest

The authors declare that the research was conducted in the absence of any commercial or financial relationships that could be construed as a potential conflict of interest.

## Publisher's Note

All claims expressed in this article are solely those of the authors and do not necessarily represent those of their affiliated organizations, or those of the publisher, the editors and the reviewers. Any product that may be evaluated in this article, or claim that may be made by its manufacturer, is not guaranteed or endorsed by the publisher.
