# Supplementary material for: Bioaccessibility and Gut Metabolism of Free and Melanoidin-Bound Phenolic Compounds From Coffee and Bread
Source: Front Nutr. 2021 Jul 26;8:708928. doi: 10.3389/fnut.2021.708928 (PMC8349987; doi:10.3389/fnut.2021.708928)
Supplement: Supplementary file 1 [file Table_1.DOCX]

**Supplementary Table 1.** Composition (for 1 L) of simulated gastric and intestinal fluids used for *in vitro* gastrointestinal digestion and of growth medium used for *in vitro* gut fermentation.

| **Gastric fluid (pH 2.0)** | | **Intestinal fluid (pH 6.5)** | | **Growth medium** | |
| --- | --- | --- | --- | --- | --- |
| NaCl (g) | 5.50 | NaCl (g) | 13.50 | Peptone (g) | 2.0 |
| NaH_2_PO_4_ (g) | 0.54 | CaCl_2_ (g) | 0.41 | Yeast extract (g) | 2.0 |
| KCl (g) | 1.64 | NaHCO_3_ (g) | 7.98 | NaHCO_3_ (g) | 2.0 |
| CaCl_2_ (g) | 0.84 | KH_2_PO_4_ (g) | 0.12 | CaCl_2_ (g) | 0.01 |
| NH_4_Cl (g) | 0.62 | KCl (g) | 1.024 | NaCl (g) | 0.1 |
| Glucose (g) | 1.30 | MgCl_2_ (g) | 0.075 | Pectin (g) | 2.0 |
| Urea (g) | 0.17 | Urea (g) | 0.275 | Xylan (g) | 2.0 |
| Mucin (g) | 6.00 | Concentrate HCl (mL) | 0.37 | Gum Arabic (g) | 2.0 |
| Bovine serum albumin (g) | 2.00 | Porcine pancreatin (g)* | 8.0 | Potato starch (g) | 5.0 |
| Concentrated HCl (mL) | 16.6 | Bile extract (g)* | 50.0 | Casein (g) | 3.0 |
| Porcine pepsin (g)* | 5.28 | Bovine serum albumin (g)* | 2.4 | Inulin (g) | 1.0 |

*Reagent added on the day of the experiment.
